# Supplementary material for: Reversal of Myofibroblast Apoptosis Resistance and Collagen Deposition by Phaseoloidin-Induced Autophagy Attenuates Pulmonary Fibrosis
Source: Biomedicines. 2025 Oct 31;13(11):2679. doi: 10.3390/biomedicines13112679 (PMC12650212; doi:10.3390/biomedicines13112679)
Supplement: Supplementary file 1 [file biomedicines-13-02679-s001.zip › biomedicines-3862406-supplementary.pdf]

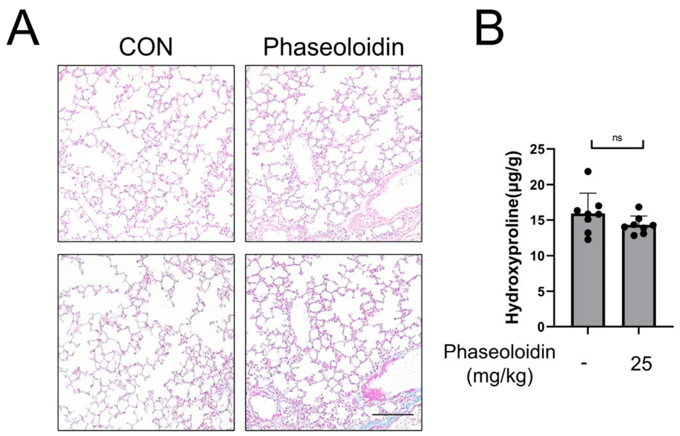

Figure S1. Phaseoloidin treatment alone. (**A**) HE and Masson staining Scale bar representing 100 μm. (**B**) Hydroxyproline content in the control group versus in the phaseoloidin-only group.
